# Precision virotherapy for IDH-mutant astrocytoma

**DOI:** 10.1016/j.omton.2026.201326

**Published:** 2026-08-28

**Authors:** Eleni Panagioti, Sean E. Lawler, Michael B. Yaffe, E. Antonio Chiocca, Charles H. Cook

**Affiliations:** 1 Department of Surgery, Beth Israel Deaconess Medical Center, Harvard Medical School, Boston, MA, USA; 2 Department of Pathology and Laboratory Medicine, Legorreta Cancer Center, Brown University, Providence, RI, USA; 3 Center for Precision Cancer Medicine, David. H. Koch Institute for Integrative Cancer Research, Massachusetts Institute of Technology, Cambridge, MA, US; 4 Broad Institute of Massachusetts Institute of Technology and Harvard, Cambridge, MA, USA; 5 Harvey Cushing Neuro-oncology Laboratories, Department of Neurosurgery, Mass General Brigham Neuroscience Institute, Harvard Medical School, Boston, MA, USA; 6 Center for Tumors of the Nervous System, Mass General Brigham Cancer Institute, Boston, MA, USA

## Main text

Despite decades of investigation, high-grade diffuse gliomas remain among the most therapeutically challenging malignancies. While molecular classification has transformed our understanding of glioma biology, relatively few genomic alterations have been translated into predictive biomarkers that guide therapeutic selection. The discovery of recurrent mutations in *isocitrate dehydrogenase 1* (*IDH1*; codon 132) or 2 (*IDH2*; codon 172) genes fundamentally redefined diffuse glioma classification, revealing profound metabolic, epigenetic, and immunological differences that may influence tumor progression.^1^ The recent approval of mutant IDH inhibitors represents an important advance; however, clinical benefit remains largely confined to a subset of patients with grade 2 diffuse gliomas, while patients with high-grade (WHO grades 3 and 4) IDH-mutant astrocytomas continue to experience disease progression.^2^ These limitations highlight the need to identify additional vulnerabilities created by mutant IDH that can be therapeutically exploited.

Oncolytic virotherapy has emerged as a promising therapeutic strategy for diffuse glioma by coupling direct tumor cell killing with activation of antitumor immunity. Clinical progress, including the approval of the herpes simplex virus type 1 (HSV-1) vector G47Δ^3^ in Japan and encouraging results with CAN-3110^4^ (formerly rQNestin34.5v.2), has renewed enthusiasm for HSV-1-based virotherapy. Nevertheless, clinical responses remain heterogeneous, highlighting the need for biomarkers that identify patients most likely to benefit. Whether molecular subtypes of diffuse glioma differ in their susceptibility to HSV-1-based virotherapy has remained largely underexplored.

**Figure 1:**
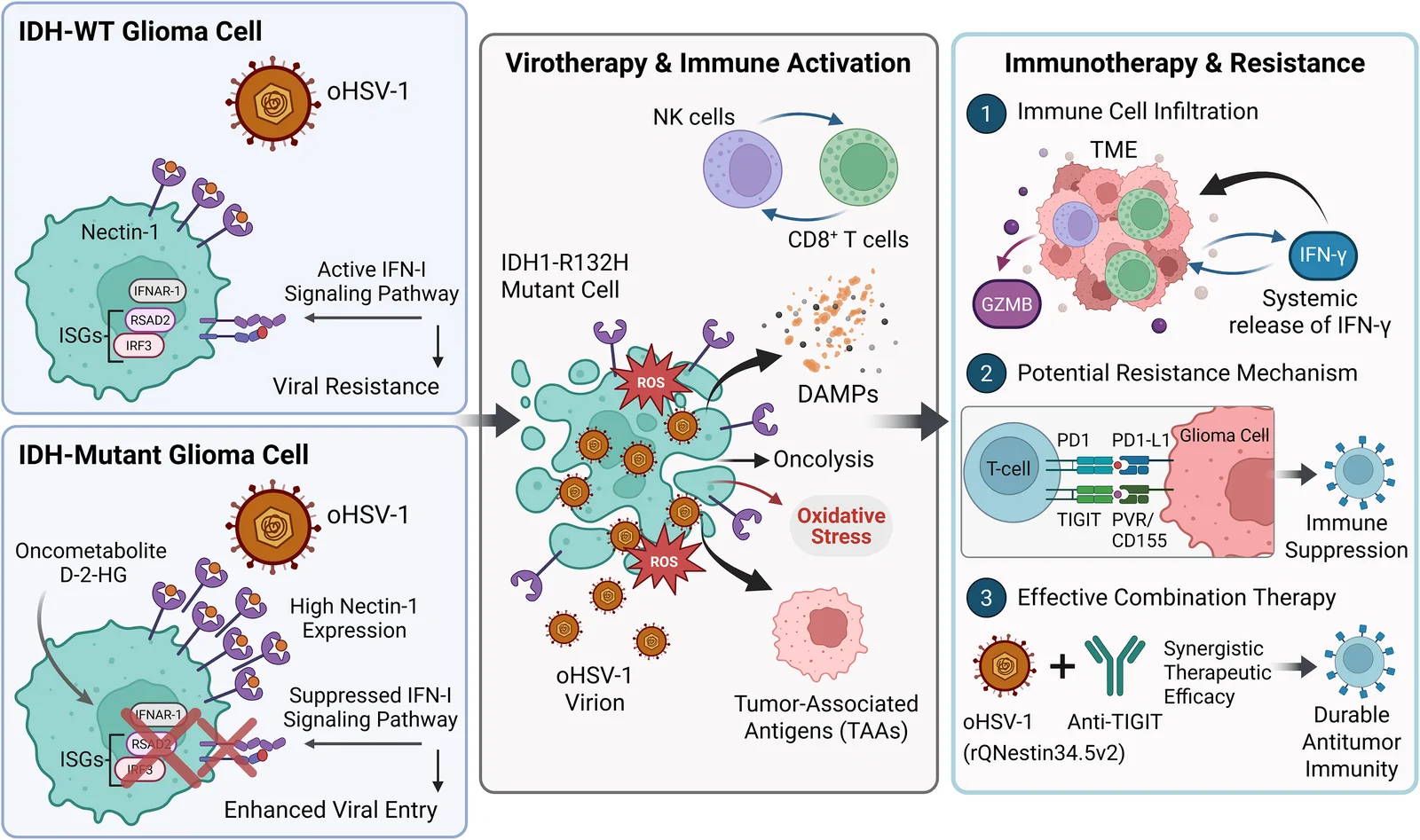
IDH1-R132H rewires high-grade astrocytoma and antitumor immunity to enhance HSV-1 immunovirotherapy Compared with IDH-wild-type tumors, IDH1-mutant astrocytomas upregulate NECTIN-1, promoting HSV-1 entry, while suppressing intrinsic antiviral type I interferon signaling, including IFNAR1 and interferon-stimulated genes (e.g., RSAD2 and IRF3). These changes establish a cellular state permissive to HSV-1 infection and efficient viral replication. Infection with oncolytic HSV-1 rQNestin34.5v.2 induces oxidative stress through the generation of reactive oxygen species (ROS), leading to immunogenic tumor cell death, the release of damage-associated molecular patterns (DAMPs), activation of innate immunity, antigen presentation, and the generation of tumor-specific adaptive immune responses. Despite enhanced therapeutic efficacy, oHSV-1 treatment also induces adaptive immune resistance through activation of the PVR/CD155-TIGIT and PD-1/PD-L1 immune checkpoint pathways, identifying rational targets for combination immunotherapy. In syngeneic murine glioma models, combining oHSV-1-rQNestin34.5v.2 with anti-TIGIT blockade synergistically induced durable antitumor immunity. Created with BioRender. Panagioti, E. (2026) b1246z4. https://BioRender.com/b1246z4.

In our recent *Nature Communications* study,^5^ we show that beyond its canonical role as a metabolic driver, the common IDH1-R132H mutation rewires multiple cellular processes to establish a virus-permissive state that sensitizes high-grade astrocytomas to HSV-1 immunovirotherapy (Figure 1). Mechanistically, this phenotype is mediated by two complementary processes. First, IDH1-R132H upregulates NECTIN-1, the major HSV-1 entry receptor in glioma cells, thereby facilitating viral entry. Notably, previous studies demonstrated that NECTIN-1 expression correlates with sensitivity to HSV-1 virotherapy in patient-derived pediatric IDH-wild-type brain tumor xenografts, providing independent support for the translational relevance of NECTIN-1 in guiding HSV-1-based therapy.^6^ Second, mutant IDH suppresses intrinsic antiviral defense programs, including type I interferon signaling, consistent with previous observations by Chen et al.,^7^ while dampening inflammatory pathways that normally restrict viral replication. Collectively, these changes promote efficient viral replication while enhancing oxidative stress and apoptosis following HSV-1 infection. Consistent with these mechanistic insights, patient-derived WHO grade 3 and 4 IDH-mutant astrocytomas exhibit coordinated suppression of pathways involved in antiviral immunity and tumor cell survival, including type I interferon, IL6-JAK-STAT3, IL2-STAT5, and hypoxia signaling. Together, these findings identify IDH1-R132H-driven cellular reprogramming as a therapeutically exploitable vulnerability for HSV-1-based virotherapy in high-grade astrocytomas.

**Figure 2:**
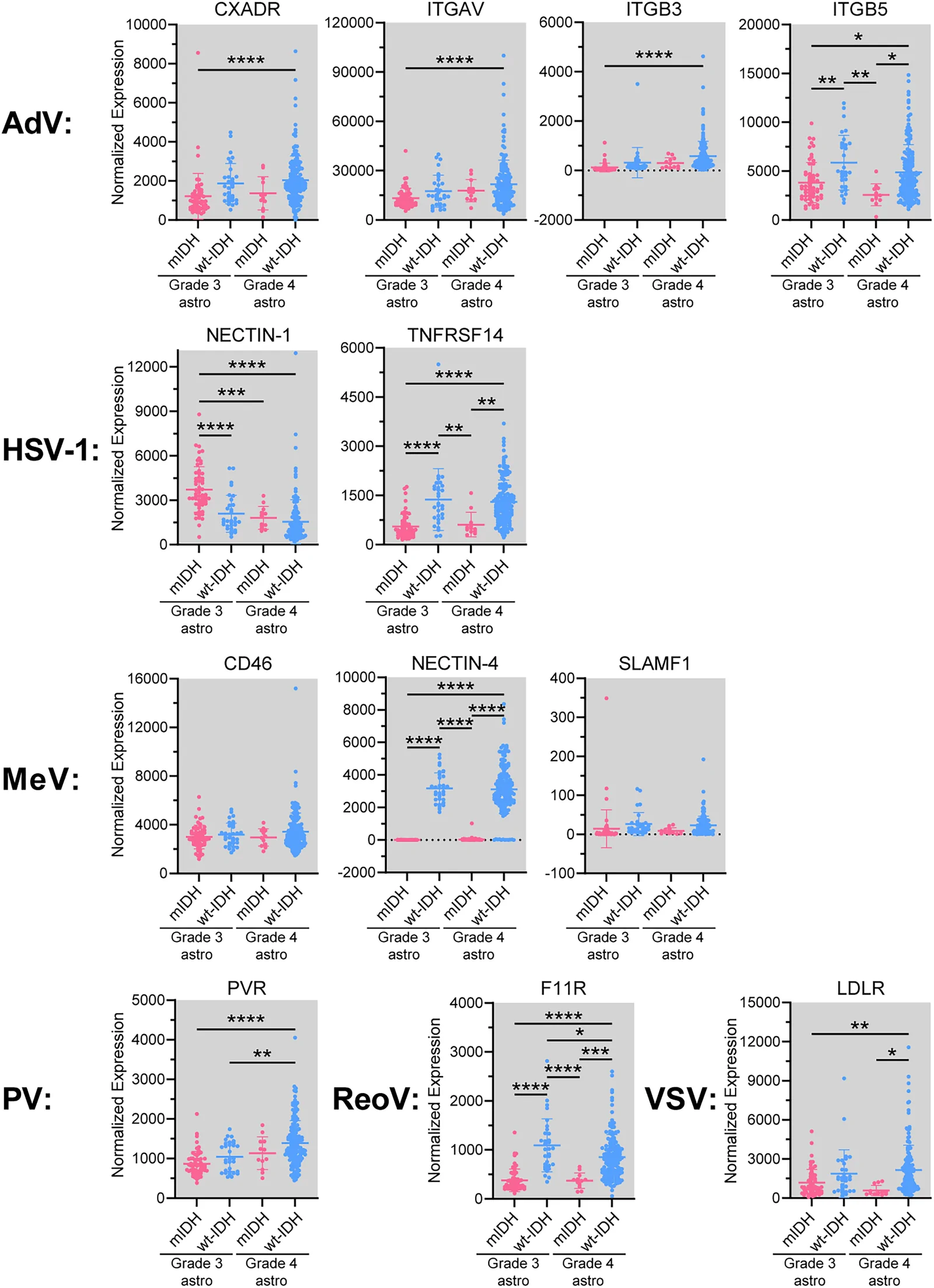
Expression of oncolytic virus entry receptors in IDH-mutant and IDH-wild-type high-grade astrocytomas Normalized expression of established viral entry receptors was analyzed in The Cancer Genome Atlas (TCGA) RNA-sequencing dataset of high-grade astrocytomas, including IDH-mutant grade 4 (*n* = 12), IDH-wild-type grade 4 (*n* = 158), IDH-mutant grade 3 (*n* = 58), and IDH-wild-type grade 3 (*n* = 31) tumors. Viral receptors evaluated included adenovirus (AdV; CXADR, ITGAV, ITGB3, ITGB5), herpes simplex virus type 1 (HSV-1; NECTIN-1, TNFRSF14/HVEM), measles virus (MeV; CD46, NECTIN-4, SLAMF1), poliovirus (PV; PVR/CD155), reovirus (ReoV; F11R), and vesicular stomatitis virus (VSV; LDLR). Data are presented as mean ± SD. Statistical significance was assessed by one-way ANOVA with Tukey’s multiple-comparisons test. Significance is indicated as *p* < 0.05, *p* < 0.01, *p* < 0.001, and *p* < 0.0001; ns, not significant (non-significant comparisons are not shown). Processed data and analysis scripts are publicly available at https://github.com/lasejour/IDH1-TCGA-Analysis, as previously described.^5^

These findings expand the role of tumor genotype as a predictive biomarker. Rather than solely determining response to targeted therapies, oncogenic mutations may also dictate susceptibility to biological therapies such as oncolytic viruses. To determine whether IDH mutations broadly influence viral entry pathways exploited by clinically tested oncolytic viruses, we analyzed expression of established viral receptors in WHO grade 3 and 4 astrocytomas from our published patient cohort^5^ (Figure 2). Receptors associated with adenovirus (CXADR, ITGAV, ITGB3, and ITGB5), measles virus (NECTIN-4), poliovirus (PVR/CD155), reovirus (F11R), and vesicular stomatitis virus (LDLR) were preferentially expressed in IDH-wild-type tumors. In contrast, NECTIN-1, the principal HSV-1 entry receptor, was the only viral receptor selectively enriched in IDH-mutant grade 3 astrocytomas. Although HVEM (TNFRSF14), another HSV-1 entry receptor, was more highly expressed in IDH-wild-type tumors, this pattern may reflect differences in immune cell composition, as HVEM is predominantly expressed by immune rather than tumor cells. These findings suggest that IDH mutations do not confer broad susceptibility to oncolytic viruses but instead create a receptor landscape selectively permissive for HSV-1 infection through increased NECTIN-1 expression. Notably, because NECTIN-1 expression declines with increasing tumor grade, IDH-mutant grade 3 astrocytomas, and potentially lower-grade gliomas, may represent an even more favorable setting for HSV-1-based virotherapy.

Beyond tumor-intrinsic mechanisms, our study identifies adaptive immune resistance as a key barrier to durable therapeutic responses. Oncolytic HSV-1 treatment induced upregulation of PVR/CD155, a highly expressed immune target in glioma, together with increased TIGIT expression on tumor-infiltrating lymphocytes, implicating activation of the PVR-TIGIT axis as a mechanism of treatment-induced immune suppression. Accordingly, combining oHSV-1-rQNestin34.5v.2 with TIGIT blockade significantly prolonged survival and generated durable tumor-specific immune memory in immunocompetent IDH1-mutant glioma models. In contrast, PD-1 blockade provided no additional benefit, suggesting that HSV-1 immunovirotherapy elicits a distinct immune resistance program. Beyond supporting TIGIT blockade as a rational combination strategy for HSV-1 virotherapy, these findings provide a clinically relevant framework for understanding immune checkpoint resistance, particularly in light of the limited clinical efficacy of combined TIGIT and PD-1 inhibition in solid tumors despite encouraging preclinical results.^8^

Host immune history may represent an additional determinant of response to HSV-1-based virotherapy. In the phase I CAN-3110 trial, durable responses were enriched among HSV-1-seropositive patients irrespective of IDH status,^4^ suggesting that baseline HSV-1 serostatus may serve as a predictive biomarker. Mechanistic analyses further demonstrated that treatment expanded pre-existing tumor-infiltrating T cell clones,^9^ indicating that a single administration of oncolytic HSV-1 can amplify antiviral immune memory to generate sustained antitumor T cell responses. Whether repeated dosing further amplifies antiviral immune memory and expands tumor-reactive T cell clonotypes remains an important question as multidose HSV-1 virotherapy is evaluated clinically (NCT03152318). These observations challenge the traditional view that pre-existing antiviral immunity is solely a barrier to viral persistence. Instead, accumulating evidence across multiple oncolytic viral platforms, including HSV-1, adenovirus, and reovirus, suggests that antiviral immune memory can function as an immunological amplifier by enhancing antigen presentation, promoting T cell activation, and strengthening antitumor immunity.^10^ Given the high prevalence of HSV-1 exposure in the population, prospective evaluation of HSV-1 serostatus together with tumor genotype, NECTIN-1 expression, interferon pathway activity, and immune profiling may enable a more personalized approach to patient selection for HSV-1-based virotherapy.

More broadly, our findings support an emerging paradigm in which oncogenic mutations shape susceptibility not only to targeted therapies but also to immunovirotherapy, opening new opportunities for genotype-guided viral immunotherapy in diffuse glioma.

## Acknowledgments

This work was supported by the 10.13039/100000002National Institutes of Health (NIH) grants U01NS061811, P01CA163205, R01NS110942, and P01CA236749 (E.A.C.); the Alliance for Cancer Gene Therapy, Greenwich, CT (E.A.C.); 10.13039/100000002NIH grant R01CA263324 (S.E.L. and C.H.C.); 10.13039/100000002NIH grant R35ES028374 (M.B.Y); CRUK Brain Tumor Award C42454/A28596 (M.B.Y.); and the Beth Israel Deaconess Medical Center 2023 Early-Stage Career Investigator Award, GRT65864 (E.P.).

## Declaration of interests

E.A.C. is a named inventor on patents related to HSV-1 rQNestin34.5v.2 and CAN-3110, which are owned by Brigham and Women’s Hospital (BWH) and licensed to Candel Therapeutics, Inc. Under this licensing agreement, BWH receives milestone payments and may receive future royalty payments. E.A.C. serves as an advisor to Bionaut Labs, Seneca Therapeutics, ReIgnite, and Calidi; holds equity options in Bionaut Labs, Seneca Therapeutics, ReIgnite, and Ternalys Therapeutics; and is a co-founder and member of the Board of Directors of Ternalys Therapeutics. E.A.C. has also received research support from the NIH, the U.S. Department of Defense, the American Brain Tumor Association, the National Brain Tumor Society, the Alliance for Cancer Gene Therapy, the Neurosurgical Research Education Foundation, Advantagene, NewLink Genetics, and Amgen.

## Declaration of generative AI and AI-assisted technologies in the writing process

During the preparation of this manuscript, the authors used ChatGPT (OpenAI) to improve the clarity and readability of the text. The authors reviewed and edited the generated content and take full responsibility for the content of the published article.
